# Local Anæsthesia

**Published:** 1854-12

**Authors:** 


LOCAL ANÆSTHESIA.—During the last two or three years, many attempts have been made to produce local anæsthesia, in order to obviate the necessity and peril of chloroform-inhalation. It is by no means improbable that these attempts will finally lead to some discovery which may be useful in surgery. We have alluded more than once to Dr. Arnott's application of cold, and we now propose to pass in review the other local measures which have been employed. Richet describes the older and recent attempts to produce local loss of sensibility by means of *ether*. The ether is simply dropped on the skin, and allowed rapidly to evaporate. Richet relates thirteen cases, in three of which tolerably deep operations were performed, viz: the excision of two small tumors, and the amputation of a toe. In the other ten cases, abscess or carbuncles were opened. Richet uses an apparatus, by means of which the ether is dropped on the part, and at the same time a current of air is directed on it, and causes rapid evaporation. Two or three minutes usually suffice. If a longer time be allowed to pass, some reaction may occur.

Ricord has applied the actual cautery to chancres in two cases after ether had been thus applied. In one case, no pain, in the other, very little was felt. In the last case, the use of the ether had been continued too long (five minutes,) and some feeling of warmth from reaction had occurred. Brochin also has opened a very tender abscess in the axilla, without giving pain, by means of the rapid evaporation of ether.—*British & For. Medico-Chir. Review.*

